# Comparison of clinical outcomes between multiple antithrombotic therapy versus left atrial appendage occlusion with dual antiplatelet therapy in patients with atrial fibrillation undergoing drug-eluting stent implantation

**DOI:** 10.1371/journal.pone.0244723

**Published:** 2021-01-07

**Authors:** Hyungdon Kook, Hee-Dong Kim, Jaemin Shim, Young-Hoon Kim, Jung-Sun Kim, Hui-Nam Pak, Hyun-Jong Lee, Rak-Kyeong Choi, Woong-Chol Kang, Eun-Seok Shin, Jai-Wun Park, Cheol Woong Yu, Do-Sun Lim

**Affiliations:** 1 Cardiovascular Center, Korea University Anam Hospital, Seoul, Korea; 2 Severance Cardiovascular Hospital, Yonsei University College of Medicine, Seoul, Korea; 3 Sejong General Hospital, Seoul, Korea; 4 Gil Hospital, Gachon University, Incheon, Korea; 5 Ulsan University Hospital, University of Ulsan College of Medicine, Ulsan, Korea; 6 Department of Cardiology, Charite University Berlin, Campus Benjamin Franklin, Berlin, Germany; CVPath Institute Inc., University of Maryland, UNITED STATES

## Abstract

**Background:**

Complex antithrombotic regimens are recommended for patients with atrial fibrillation (AF) undergoing drug-eluting stent (DES) implantation but carry high bleeding risk.

**Hypothesis:**

We aimed to evaluate whether left atrial appendage occlusion (LAAO) with dual antiplatelet therapy (DAPT) improve clinical outcomes when compared with multiple antithrombotic therapy (MAT) in patients with AF undergoing DES implantation.

**Methods:**

Among 475 AF patients who underwent DES, 41 patients treated by LAAO with DAPT and 434 patients on MAT were compared. MAT was defined as any combination of warfarin-based antithrombotic therapy. Among the MAT group, 34.8% were on triple antithrombotic therapy. The primary endpoint was a net adverse clinical event (NACE), a composite of cerebrovascular accident (CVA) and major bleeding. Secondary endpoints were CVA, major bleeding, major adverse cardiac and cerebral event (MACCE), MI, cardiovascular death, and all-cause death. Additional analysis between the new oral anticoagulant (NOAC)-based antithrombotic therapy group (n = 45) and the LAAO group was performed for the same endpoints. To adjust the confounding factors, inverse probability of treatment weighting (IPTW) was applied during the endpoint analysis.

**Results:**

The LAAO group showed higher incidences of diabetes mellitus, prior CVA, higher CHA2DS2-VASc score (4.56±1.55 vs. 2.96±1.60; P<0.0001), and higher HAS-BLED score (3.24±1.20 vs. 2.13±0.75; P<0.0001). NACE occurred less frequently in the LAAO group than the MAT group at 24 months (9.4% vs. 15.3%; hazard ratio 0.274; 95% confidence interval 0.136 – 0.553; P = 0.0003), mainly driven by the reduction in major bleeding (2.4% vs. 9.3%; hazard ratio 0.119; 95% confidence interval 0.032 – 0.438; P = 0.001). The LAAO group with greater thrombotic and hemorrhagic risks showed comparable primary/secondary outcomes with the NOAC-based anti-thrombotic therapy group.

**Conclusions:**

Among patients with AF who underwent DES implantation, the LAAO group had better net clinical outcomes for preventing CVA and major bleeding than the MAT group. Further large-scale trials including comparisons with NOACs are warranted.

## Introduction

The number of patients with simultaneous atrial fibrillation (AF) and coronary artery disease who require both antiplatelet and anticoagulation therapy is increasing in the aging society [1–3]. According to the previous report, up to 15% of patients with AF previously suffered myocardial infarction (MI) or ischemic heart disease [4]. In addition, 6-8% of patients undergoing percutaneous coronary intervention (PCI) had an indication for oral anticoagulation (OAC) [5].

Current guidelines recommend complex regimens including triple antithrombotic therapy (TAT) for patients undergoing PCI with AF [6, 7]. But the harms of TAT treatment, which may occur bleeding, are thought to outweigh prevention of thromboembolism [5]. In a Danish registry, major bleeding events occur primarily during the early period of medication, mostly within 3 months from the onset of TAT [2, 4]. In the WOEST trial, which detected a high incidence of bleeding in patients treated with TAT, most bleeding events were also concentrated during the 2 months after randomization [8].

In patients who require both OAC and antiplatelet therapy, left atrial appendage occlusion (LAAO) may have the potential benefit of requiring only antiplatelet agents without OAC. However, studies comparing LAAO combined with dual antiplatelet therapy (DAPT) versus conventional antithrombotic therapy in this setting are insufficient.

In the present study, we sought to compare the clinical outcomes of LAAO with DAPT versus multiple antithrombotic therapy (MAT) in patients with AF undergoing drug-eluting stent (DES) implantation.

## Methods

### Study population

**Fig 1:**
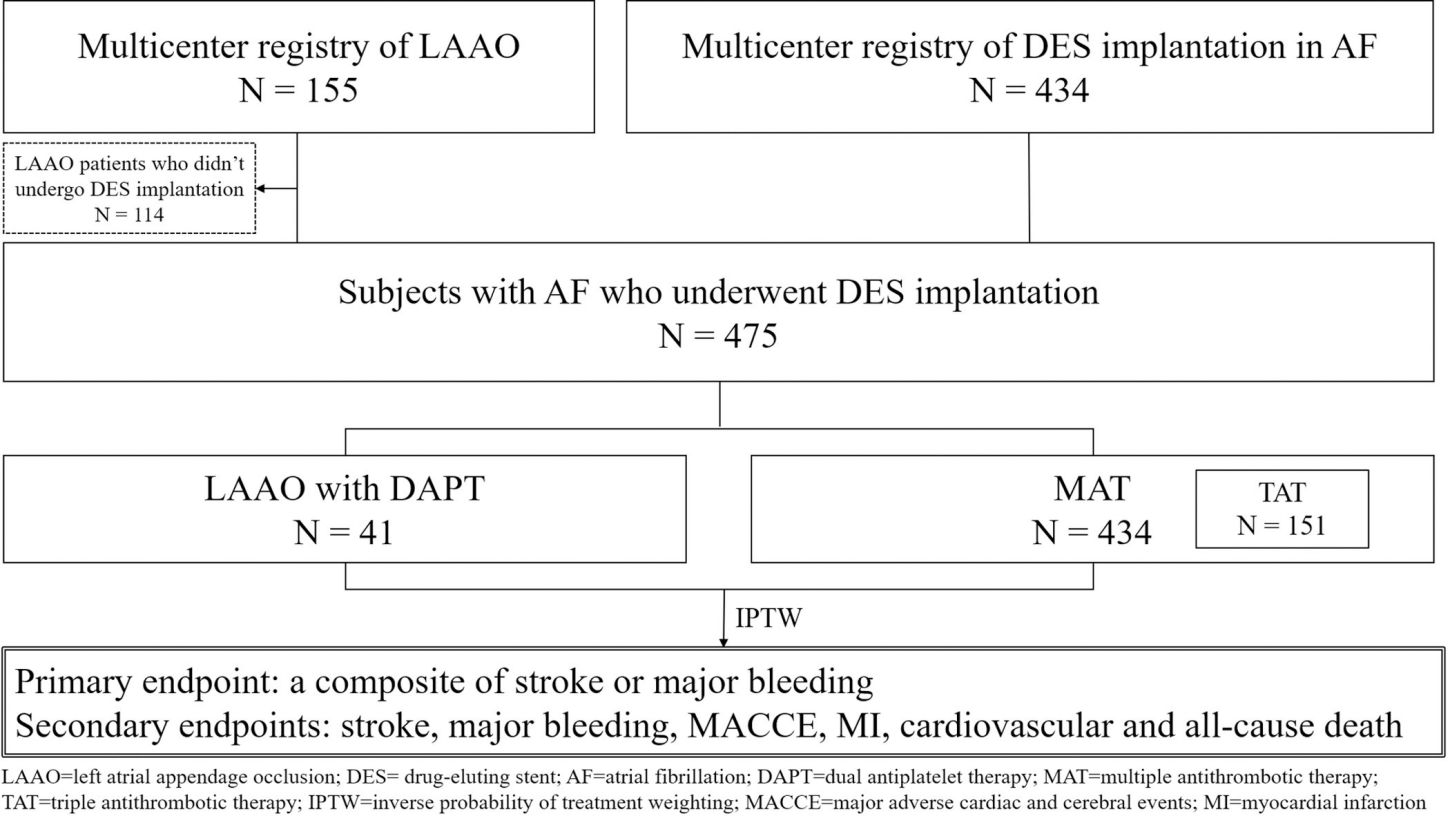
Flowchart of patient selection.

The patient selection flow chart is shown in Fig 1. We identified 434 consecutive non-valvular AF patients who underwent DES implantation in the PCI registry of Korea University Anam Hospital and Sejong General Hospital from June 2003 to February 2014 and defined these patients as the MAT group. This group of patients was treated with warfarin-based antithrombotic therapy. We also identified 41 patients who underwent DES implantation among 155 consecutive patients in a Korean multicenter LAAO registry including 5 hospitals (Korea University Anam Hospital, Sejong General Hospital, Yonsei University Severance Hospital, Gachon University Gil Hospital, and Ulsan University Hospital) from October 2010 to August 2016 as the LAAO group. We additionally identified 45 AF patients who underwent PCI and treated with new oral anticoagulant (NOAC)-based antithrombotic therapy (NAT) from October 2013 to July 2020 as the NAT group for further comparison with the LAAO group. The study protocol was approved by the Institutional Review Board of Korea University Anam Hospital (2017AN0289). The informed consent was waived.

### Definitions and endpoints

MAT was defined as any combination of warfarin-based anticoagulation or antiplatelet therapy at discharge after the DES implantation. TAT was a subgroup of MAT defined as a combination of warfarin and DAPT. Time in therapeutic range (TTR) was defined as the proportion of the time that international normalized ratio value remained between 2.0 and 3.0 according to the Rosendaal method [9]. NAT was defined as any combination of NOAC-based anticoagulation or antiplatelet therapy at discharge after the DES implantation.

The primary endpoint was a net adverse clinical event (NACE), a composite of cerebrovascular accident (CVA) and major bleeding. CVA was defined as an ischemic cerebrovascular event including both transient ischemic attack and ischemic cerebral infarction. Major bleeding event was defined as a moderate or severe bleeding according to Global Utilization of Streptokinase and Tissue Plasminogen Activator for Occluded Coronary Arteries criteria (GUSTO) [10]. In addition to GUSTO moderate or severe bleeding analysis, additional analysis was conducted for events corresponding to Bleeding Academic Research Consortium (BARC) type 3 or 5 bleeding [11].

Secondary endpoints were CVA, major bleeding, major adverse cardiac and cerebral event (MACCE), MI, cardiovascular death, and all-cause death. All endpoints were further analyzed in the comparison between the LAAO group and the TAT subgroup, and the comparison between the LAAO group and the NAT group. MACCE was defined as a quadruple composite of all-cause death, non-fatal MI, repeat revascularization, and CVA.

All endpoints were analyzed over a 24-month follow-up period. CVA were adjudicated by neurologists. All other outcomes were adjudicated by the attending physicians.

### Medical treatment and procedure protocol

The MAT group and the NAT group were composed of various subgroups. Selection of treatment regimen was left to the discretion of the attending physician. Duration of regimen, TTR, and de-escalation strategy were specified.

LAAO was indicated when patients had non-valvular AF with CHA2DS2-VASc≥2 and simultaneously had high risk of bleeding according to HAS-BLED score≥3 or the following contraindications of anticoagulation: 1) major bleeding event that occurred during anticoagulation or 2) CVA during anticoagulation. The appendage dimension and anatomy were measured using either computed tomography images or transesophageal echocardiography and fluoroscopy. Device type was chosen according to the appendage morphology. Device size was 10-20% larger than the diameter of the orifice to minimize post-procedural leakage. Implant success was defined as confirmation of the device-specified release criteria and successful device release. Peri-device leakage was defined as a residual leak >5mm observed by echocardiography.

### Statistics

Continuous variables were expressed as mean ± standard deviation. Categorical variables were expressed as frequency (percentage). Differences between the groups were analyzed using Student’s t-test for continuous variables and Pearson’s χ2-test or Fisher’s exact test for categorical variables. To adjust the confounding factors, inverse probability of treatment weighting (IPTW) was applied during the endpoint analysis. Multilevel propensity score was calculated as the probability of receiving LAAO based on the following variables: age, sex, smoking, dyslipidemia, hypertension, diabetes mellitus, congestive heart failure, previous history of CVA, ejection fraction, type of AF, lesion number of the coronary artery disease, and number and diameter of implanted stents. Additional comparisons of the study endpoints between the LAAO group and the TAT subgroup, and between the LAAO group and the NAT group were performed after the independent IPTW adjustment using the same covariates mentioned above. We analyzed the cumulative incidence rate from the index procedure time to the time of the first clinical outcome of interest, estimated using the Kaplan–Meier method with log-rank test. We used the Cox multivariate model incorporating covariates with P-value< 0.1 after IPTW adjustment to examine the hazard ratio (HR) and 95% confidence interval (CI) of each endpoint. All tests were two-tailed, with P-value<0.05 was considered significant. Statistical analysis was carried out using the R statistical computing environment ver. 3.3.2 (R Development Core Team).

## Results

### Baseline characteristics

Baseline characteristics are shown in Table 1. Compared to the MAT group (n = 434), the LAAO group (n = 41) was more likely to have diabetes mellitus, previous CVA, higher mean CHA2DS2-VASc score (4.56±1.55 vs. 2.96±1.60; P<0.0001), and higher mean HAS-BLED score (3.24±1.20 vs. 2.13±0.75; P<0.0001). Follow-up duration was shorter in the LAAO group. Most patients were treated with clopidogrel among P2Y12 inhibitors (80.5% in the LAAO group, 96.8% in the MAT group). Although the clinical presentation of acute coronary syndrome at index PCI was more frequent in the MAT group (46.4% in the LAAO group, 74.2% in the MAT group), the use of potent P2Y12 inhibitors such as ticagrelor and prasugrel was rarer in the MAT group. IPTW-adjusted baseline characteristics are shown in S1 Table in S1 File. Among the MAT group patients, 60.4% were treated with only DAPT without anticoagulation at discharge, followed by TAT (34.8%) (Table 2). In the TAT subgroup, mean TTR was low at 25.4%, and mean TAT duration was longer than 1 year. TAT regimens were mostly de-escalated to DAPT or double antithrombotic therapy. Baseline characteristics comparison between the LAAO group and the NAT group are shown in S2 Table in S1 File. The NAT group were older (69.6±8.3 vs. 73.9±9.7; P = 0.021) and had fewer incidence of chronic heart failure, dyslipidemia, and prior history of CVA. CHADS2 score, CHA2DS2-VASc score (4.56±1.55 vs. 3.24±1.43; P<0.001), and HAS-BLED score (3.24±1.20 vs. 2.20±0.87; P<0.001) was significantly lower in the NAT group. Among the NAT group, most of patients (80.0%) were prescribed with TAT, and the mean duration of TAT was about 4 months (S3 Table in S1 File).

**Table 1:** Baseline characteristics of the study population.

	Total (n = 475)	LAAO (n = 41)	MAT (n = 434)	P-value
Age (years)	68.0±9.2	69.6±8.3	67.9±9.2	0.2529
Age>65	329 (69.3)	28 (68.3)	301 (69.4)	0.8880
Age>75	100 (21.1)	11 (26.8)	89 (20.5)	0.3425
Male	318 (67.0)	32 (78.1)	286 (65.9)	0.1139
Follow-up period (days)	1255.2±906.1	590.5±420.3	1318.0±914.7	<0.0001
Smoking				0.0014
Never	292 (61.5)	36 (87.8)	256 (59.0)	
Ex-smoker	112 (23.6)	3 (7.3)	109 (25.1)	
Current smoker	71 (15.0)	2 (4.9)	69 (15.9)	
HTN	358 (75.4)	29 (70.7)	329 (75.8)	0.4710
DM	149 (31.4)	20 (48.8)	129 (29.7)	0.0119
CHF	137 (28.8)	15 (36.6)	122 (28.1)	0.2522
Dyslipidemia	223 (47.0)	24 (58.5)	199 (45.9)	0.1198
Previous CVA	81 (17.1)	18 (43.9)	63 (14.5)	<0.0001
CHADS2 score	1.91±1.22	2.71±1.27	1.83±1.19	<0.0001
CHA2DS2-VASc score	3.10±1.66	4.56±1.55	2.96±1.60	<0.0001
CHA2DS2-VASc≥2	391 (82.3%)	41 (100.0)	350 (80.6)	<0.0001
HAS-BLED score	2.23±0.85	3.24±1.20	2.13±0.75	<0.0001
EF (%)	52.13±13.26	54.35±11.01	51.92±13.45	0.2621
EF<40%	96 (20.2)	5 (12.2)	91 (21.0)	0.1812
Lesion number	2.11±1.06	2.00±0.86	2.12±1.06	0.6336
Stent number	1.54±0.81	1.28±0.51	1.57±0.83	0.0018
Diameter (mm)	2.97±0.43	3.14±0.50	2.92±0.42	0.0093
Length (mm)	34.79±22.27	22.00±7.78	35.97±22.79	<0.0001
AF type				0.6112
Paroxysmal	132 (27.8)	10 (24.4)	122 (28.1)	
Persistent or permanent	343 (72.2)	31 (75.6)	312 (71.9)	
Clinical presentation at index PCI				0.004
AMI	104 (21.9)	4 (9.8)	100 (23.0)	
Unstable angina	237 (49.9)	15 (36.6)	222 (51.2)	
Stable angina	110 (23.2)	11 (26.8)	99 (22.8)	
ICMP	15 (3.2)	7 (17.1)	8 (1.8)	
Silent myocardial ischemia	9 (1.9)	4 (9.8)	5 (1.2)	
P2Y12 inhibitors				0.183
Clopidogrel	453 (95.4)	33 (80.5)	420 (96.8)	
Ticagrelor	11 (2.3)	4 (9.8)	7 (1.6)	
Prasugrel	4 (0.8)	4 (9.8)	-	
Miscellaneous	7 (1.5)	-	7 (1.6)	

Values are mean ± SD or n (%). AF = atrial fibrillation; AMI = acute myocardial infarction; CHD = coronary heart disease; CHF = congestive heart failure; CVA = cerebrovascular accidents; DM = diabetes mellitus; EF = ejection fraction; HTN = hypertension; ICMP = ischemic cardiomyopathy; LAAO = left atrial appendage occlusion; MAT = multiple antithrombotic therapy; PCI = percutaneous coronary intervention.

**Table 2:** Composition of the multiple antithrombotic therapy group.

Variables	n = 434
DAPT	262 (60.4)
TAT	151 (34.8)
Duration (Days)	398.57±660.13
Time in therapeutic range (%)	25.36±23.49
De-escalation after TAT	
DAPT	43 (28.5)
Double antithrombotic therapy	41 (27.2)
Miscellaneous	3 (2.0)
Not available	64 (42.4)
Miscellaneous (Double or quadruple antithrombotic therapy)	21 (4.8)

Values are mean ± SD or n (%). DAPT = dual antiplatelet therapy; TAT = triple antithrombotic therapy.

### Procedure-related outcomes

Procedural characteristics of the LAAO group are summarized in Table 3. Two types of devices, Amplatzer™ cardiac plug or Watchman™, were equally frequently used. The device success rate was 92.7%. Among 3 cases of LAAO failure, 2 were due to size-mismatch, and 1 failed due to appendage perforation. The complication rate of the procedure was 9.8%. No procedure-related deaths occurred. One case of perforation during wiring into the appendage resulted in conversion to surgery.

**Table 3:** Procedural characteristics in the left atrial appendage occlusion group.

Variables	n = 41
LA diameter (mm)	48.44±8.44
LA volume index (ml/m^2^)	52.18±24.83
LAA type	
Chicken wing	7 (17.1)
Wind sock	8 (19.5)
Cauliflower	3 (7.3)
Cactus	4 (9.8)
Not available	19 (46.3)
LAA diameter (mm)	24.46±4.60
Perimeter (mm)	26.79±2.10
Area (mm^2^)	84.13±6.59
LAA depth (mm)	
45˚	20.23±3.56
90˚	21.04±4.14
135˚	22.39±4.58
LAA emptying velocity (cm/sec)	23.29±19.14
SEC	22 (53.7)
Thrombus	3 (7.3)
Device type	
ACP or ACP II	20 (48.8)
Watchman™	21 (51.2)
Device size (mm)	25.77±2.83
Successful implantation	38 (92.7)
Peri-device leakage	0 (0)
Complications	4 (9.8)
Pericardial effusion	1 (2.4)
Cardiac tamponade	1 (2.4)
LAA perforation	1 (2.4)
Vascular access site bleeding	1 (2.4)

Values are mean ± SD or n (%). ACP = Amplatzer™ cardiac plug; LA = left atrium; LAA = left atrial appendage; LAAO = left atrial appendage occlusion; SEC = spontaneous echo contrast.

### Clinical outcomes

**Fig 2:**
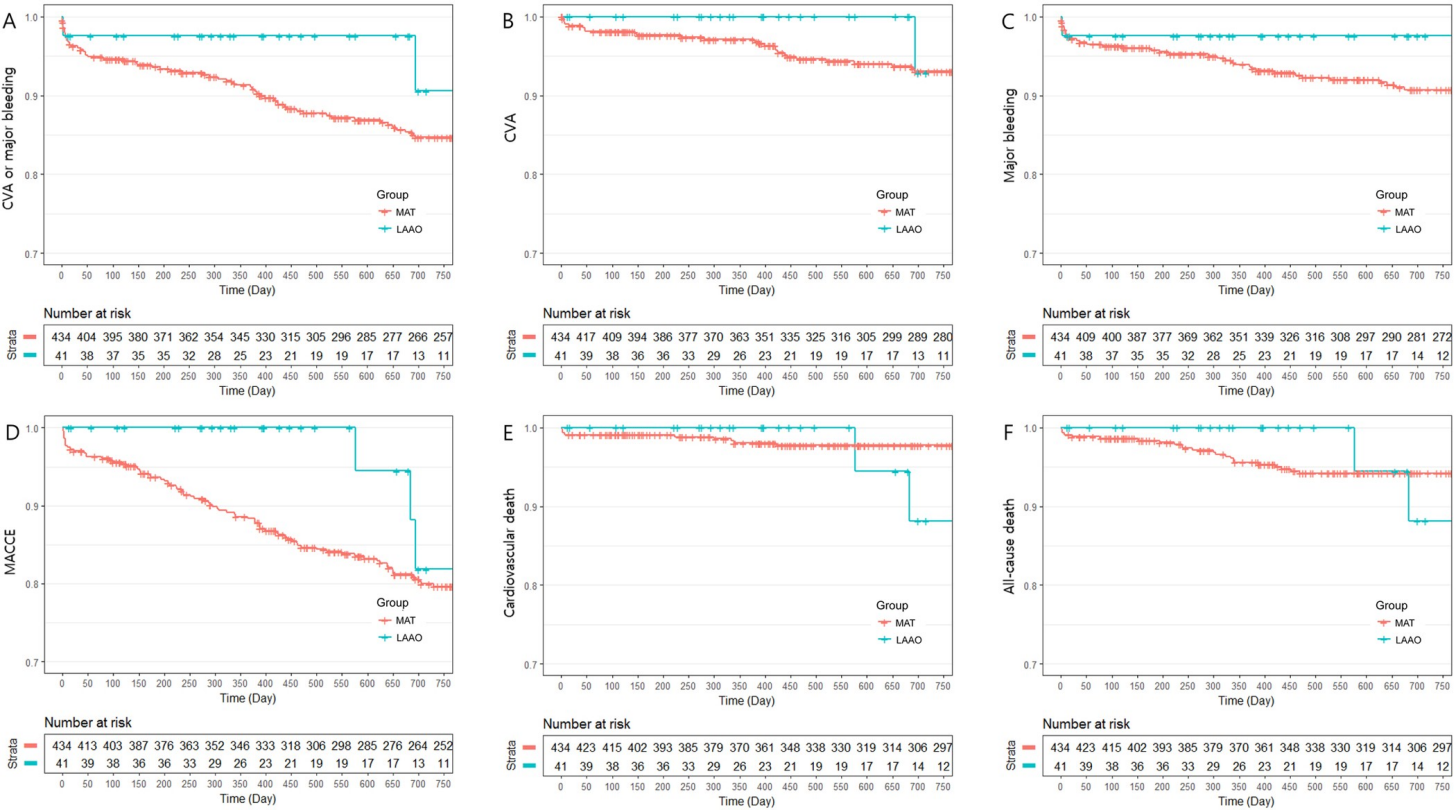
Kaplan Meier curve: Freedom from clinical outcomes between the left atrial appendage occlusion group and the multiple antithrombotic therapy group during 24 months of follow-up. A: Rates of composite of cerebrovascular accidents (CVA) or major bleeding; B: Rates of CVA; C: Rates of major bleeding; D: Rates of major adverse cardiac and cerebral events (MACCE); E: Rates of cardiovascular death; F: Rates of all-cause death.

**Fig 3:**
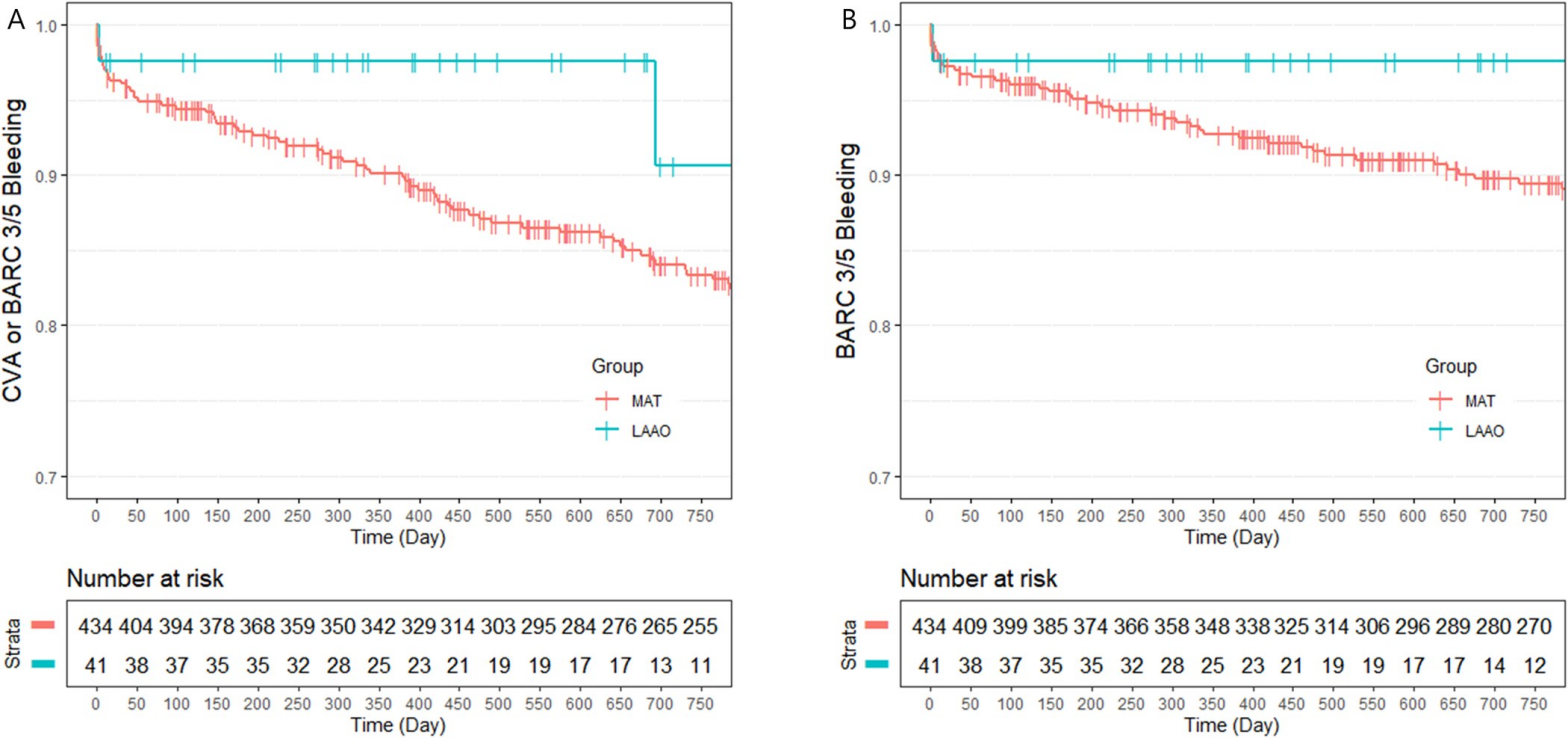
Kaplan Meier curve: Freedom from clinical outcomes using Bleeding Academic Research Consortium (BARC) definition for bleeding between the left atrial appendage occlusion group and the multiple antithrombotic therapy group during 24 months of follow-up. A: Rates of composite of cerebrovascular accidents (CVA) or BARC type 3/5 bleeding; B: Rates of BARC type 3/5 bleeding.

IPTW-adjusted clinical outcomes between the LAAO group and the MAT group are summarized in Table 4. The primary endpoint, composite of CVA and major bleeding, occurred significantly less often in the LAAO group (9.4% vs. 15.3%; HR 0.274; 95% CI 0.136 – 0.553; P = 0.0003, Fig 2). Similar result was shown in the analysis for the composite endpoint of CVA and BARC type 3 or 5 bleeding (4.9% vs. 14.1%; HR 0.191; 95% CI 0.037 – 0.992; P = 0.049, Fig 3). A beneficial but not significant trend was observed in the LAAO group regarding the CVA rate. Major bleeding events (2.4% vs. 9.3%; HR 0.119; 95% CI 0.032 – 0.438; P = 0.001), BARC type 3 or 5 bleeding events (2.4% vs. 9.4%; HR 0.082; 95% CI 0.010 – 0.657; P = 0.019), and MACCE (18.1% vs. 20.4%; HR 0.383; 95% CI 0.218 – 0.673; P = 0.001) were also less frequently shown in the LAAO group. The two cardiovascular deaths in the LAAO group occurred more than 1 year after the index procedure and were due to MI and sudden cardiac death. These deaths contributed to the significant differences observed between the two groups (11.9% vs. 2.2%; HR 3.813; 95% CI 1.297 – 11.209; P = 0.015). There were no significant differences between the LAAO group and the MAT group regarding all-cause death. Similar results were observed in comparison between the LAAO group and the TAT subgroup (S4 Table in S1 File). The incidence of NACE was lower in the LAAO group than the TAT subgroup (9.4% vs. 19.0%; HR 0.34; 95% CI 0.146 – 0.795; P = 0.013, S1 Fig). However, there was no difference in MACCE between the LAAO group and the TAT subgroup. The comparison between the non-TAT subgroup and the LAAO group also showed a similar trend to the MAT group and the LAAO group (S2 Fig and S5 Table in S1 File). The comparison between the NAT group and the LAAO group regarding primary endpoint and secondary endpoints showed no statistical differences (S3 Fig and S6 Table in S1 File).

**Table 4:** Inverse probability of treatment weighting -adjusted clinical outcomes at 24 months of follow-up.

Variables	LAAO (n = 41)	MAT (n = 434)	Hazard ratio (95% Confidence interval)	P-value
CVA or major bleeding	2 (9.4%)	59 (15.3%)	0.274 (0.136 – 0.553)	0.0003
CVA	1 (7.1%)	26 (6.9%)	0.493 (0.208 – 1.17)	0.109
Major bleeding	1 (2.4%)	36 (9.3%)	0.119 (0.032 – 0.438)	0.001
CVA or BARC type 3/5 bleeding	2 (4.9%)	61 (14.1%)	0.191 (0.037 – 0.992)	0.049
BARC type 3/5 bleeding	1 (2.4%)	41 (9.4%)	0.082 (0.010 – 0.657)	0.019
Myocardial infarction	1 (5.6%)	11 (2.9%)	0.251 (0.047 – 1.345)	0.107
MACCE	3 (18.1%)	79 (20.4%)	0.383 (0.218 – 0.673)	0.001
Cardiovascular death	2 (11.9%)	9 (2.2%)	3.813 (1.297 – 11.209)	0.015
All-cause death	2 (11.9%)	23 (5.8%)	0.915 (0.401 – 2.088)	0.833

Data shown are number of patients (%). Event rates are Kaplan-Meier estimates. Adjusted variables for Cox analysis were smoking, diabetes mellitus, ejection fraction, stent number, and stent length. BARC = Bleeding Academic Research Consortium; CVA = cerebrovascular accidents; DAPT = dual antiplatelet therapy; LAAO = left atrial appendage occlusion; MACCE = major adverse cardiac and cerebral event; MAT = multiple antithrombotic therapy.

### Relative risk reductions of CVA and major bleeding

**Fig 4:**
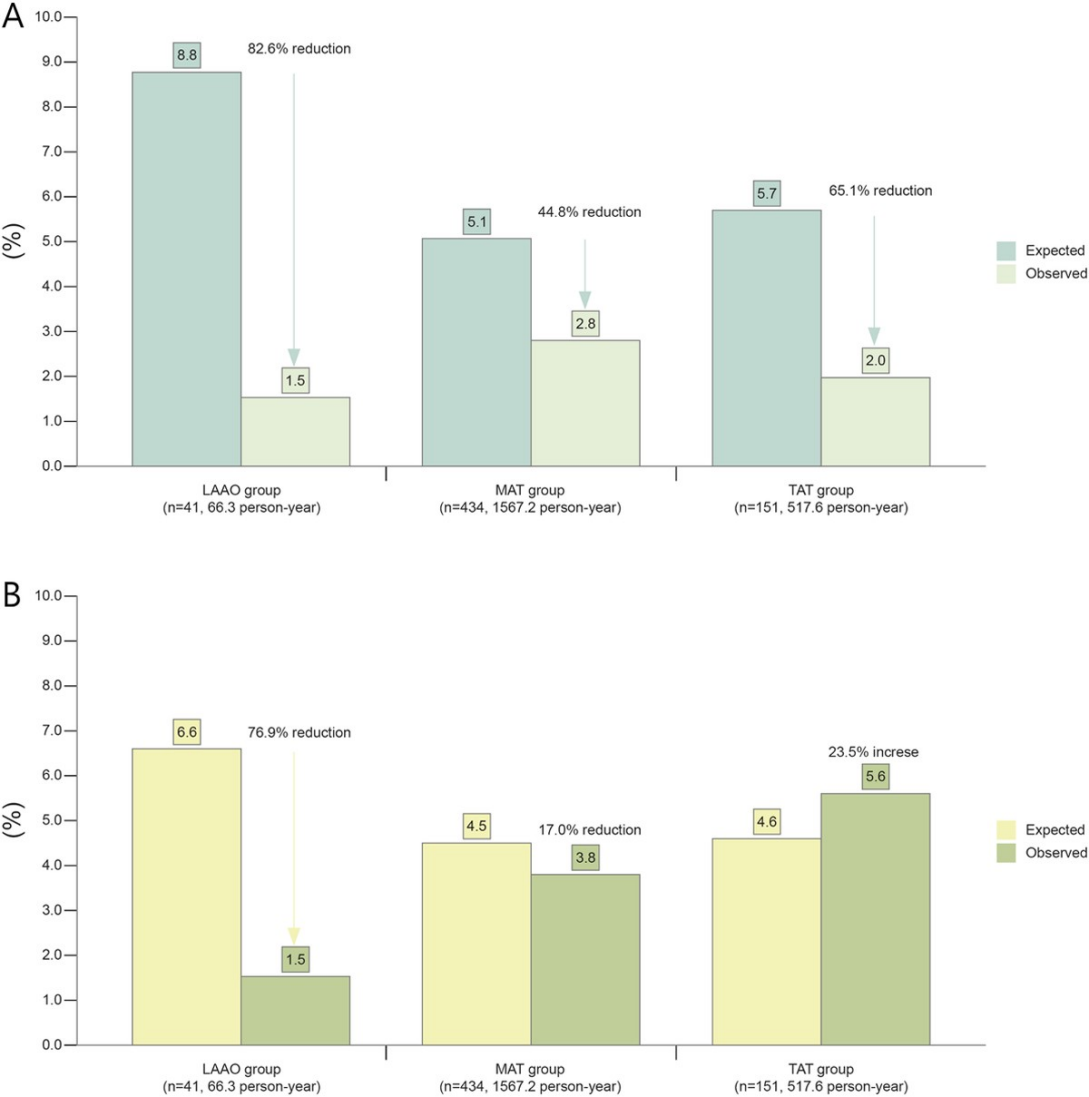
Expected and observed CVA and bleeding events according to study group. Expected rates of CVA (A) and bleeding events (B) based on CHA2DS2-VASc and HAS-BLED scores were compared with observed event rates.

During a 66.3-person-year follow-up, the actual annual rate of CVA and major bleeding was 1.54 events per 100 patient-years in the LAAO group (Fig 4). The expected annual rates of relative risk reduction (RRR) calculated using CHA2DS2-VASc and HAS-BLED scores were 82.6% for CVA and 76.9% for major bleeding, respectively. In the MAT group, RRR was 44.8% and 17.0%, respectively. In the TAT subgroup, RRR of CVA was 65.1%, but major bleeding was 23.5% higher than expected. In the non-TAT subgroup, RRR of CVA was 36.1% and RRR of major bleeding was 39.2% (S4 Fig).

### Subgroup analysis

**Fig 5:**
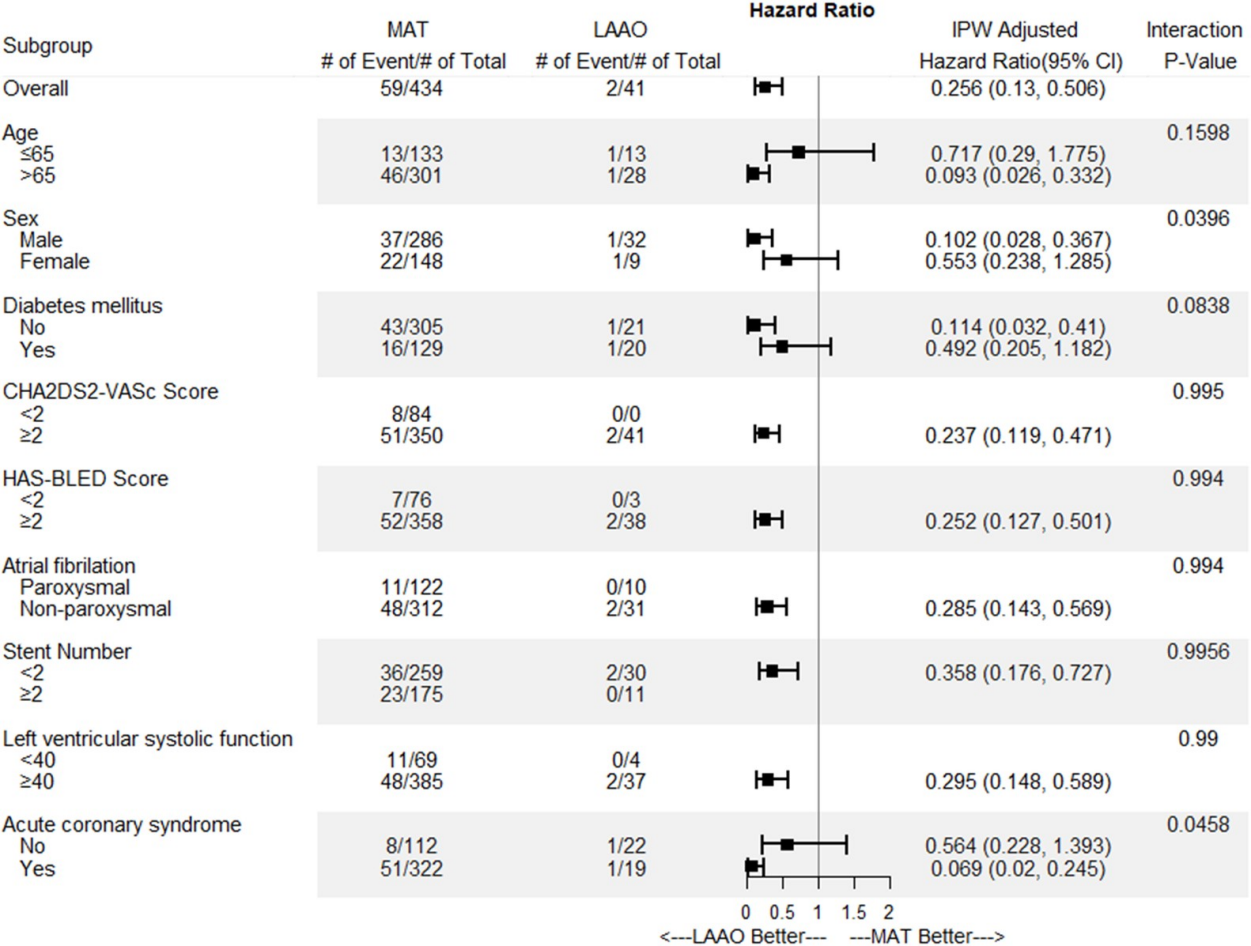
Subgroup analysis plot of the primary endpoint between the left atrial appendage occlusion group and the multiple antithrombotic therapy group.

In subgroup analyses of the primary endpoint at 24-months involving comparisons between the LAAO group and the MAT group, the LAAO group had better outcomes in terms of preventing CVA and major bleeding across the most subgroups (Fig 5). However, patients younger than 65 years, female patients, patients with diabetes mellitus, and patients without acute coronary syndrome experienced neutral effects associated with LAAO.

## Discussion

### Study findings

This study demonstrated that LAAO with DAPT resulted in net clinical benefit in terms of preventing both CVA and major bleeding compared with MAT in patients with AF undergoing DES implantation. This result was mainly driven by the reduction of major bleeding, which suggests that LAAO may simultaneously reduce bleeding risk and prevent CVA, given that the bleeding risk associated with OAC could be avoided by LAAO. The clinical benefit of the LAAO group was consistently shown in the analysis using the BARC bleeding criteria instead of the GUSTO bleeding criteria. Major bleeding events were most frequent (13.24% at 24 months) in the TAT subgroup. We also obtained an acceptable success rate of 92.7% and complication rate of 9.8% for the LAAO procedure, rates comparable with those reported in recent global studies [12, 13]. TAT was substantially under-prescribed in the MAT group, and the mean TTR value was low. These findings are in line with those of previous studies, which demonstrated underuse of TAT and low TTR rates of warfarin [14, 15]. This study also found poor compliance with adequate de-escalation of the regimen, as in the previous study [16]. The LAAO group with greater thrombotic and hemorrhagic risks showed comparable clinical outcomes with the NAT group in the present study. However, since the number of subjects was quite limited in our study, the results of the present study should be reconfirmed in large-scale studies.

### Pitfalls of conventional antithrombotic therapy

The latest European guidelines recommend to treat AF patients who are in need of OAC with short-term TAT (1 week to 1 month) after PCI according to ischemic, thrombotic, and bleeding risk and with sequential double therapy with OAC and aspirin or clopidogrel for a few months, followed by lifelong OAC monotherapy [6, 7]. However, these regimens include diverse de-escalating strategies and are very complex for both physicians and patients. In addition, these regimens may substantially increase bleeding risk, especially during the early TAT period [2, 8]. Recent studies have demonstrated that both average daily bleeding rates and average daily ischemic rates are highest during the first month immediately after PCI, followed by a gentle downward curve [17, 18]. These high bleeding rate and ischemic rate in the first month after the PCI is estimated to be even higher in AF patients undergoing PCI. Therefore, during the early period after PCI, where both average daily bleeding rates and average daily ischemic rates are high, LAAO, which may prevent both bleeding and thromboembolism, is expected to be useful in this particular patient population.

Recent trials have demonstrated the safety of new antithrombotic regimens including NOAC, their efficacy has not been fully evaluated and these trials excluded patients with advanced chronic kidney disease [19–21]. Unfortunately, with relatively small study subjects, patients with advanced chronic kidney disease who are not eligible for NOAC treatment (chronic kidney disease> = stage 4 15/475, 3.2% of total study population) were very rare, thereby conducting subgroup analysis was not applicable in our study. Future studies should be discussed further focusing on this special situation.

In addition, vitamin K antagonist has been used for preventing thromboembolic events in patients with non-valvular AF patients for over 60 years [22]. Thus, it is expected that a significant number of non-valvular AF patients are still being treated with warfarin. Also, with the aging of society, the number of patients who cannot be treated with NOAC, such as patients with advanced chronic kidney disease is increasing significantly. We believe that the results of this study can be a meaningful reference for patients who need to maintain warfarin, not NOAC, that is, patients with valvular AF or mechanical valve replacement.

### Combinations of LAAO with DAPT

It is well known that LAAO shows a CVA prevention effect equivalent to that of anticoagulation but is associated with several procedure-related complications [23–25]. Therefore, the benefits and risks of LAAO for AF patients undergoing DES implantation should be addressed before widespread usage. Although complication rates ranged from 8% to 12% during the early period after LAAO introduction, they have rapidly decreased to 4% [12, 26]. Therefore, if LAAO is performed by experienced physicians, it is thought to be safe [12, 27]. In addition, LAAO with DAPT strategy may have additional potential strengths when used to treat patients with AF undergoing DES implantation.

First, patients may be free of OAC after LAAO procedure, thereby avoiding the high risk of bleeding associated with the early TAT period [28]. Furthermore, serial monitoring of TTR of warfarin is not necessary. New potent P2Y12 inhibitors such as ticagrelor or prasugrel are also available for use in this novel treatment strategy [29].

Second, antithrombotic regimens prescribed after PCI for patients with AF could be simplified to DAPT only. Treatment with LAAO is therefore expected to improve compliance.

Third, this treatment strategy will be of great value for treating patients with advanced chronic kidney disease who are at high bleeding risk and in whom the use of NOAC is contraindicated.

The Munich consensus suggested that LAAO is a potential indication for PCI in patients with AF [30], and the results of the present study support that suggestion. Based on this background, two prospective studies comparing LAAO with DAPT and antithrombotic therapy including NOACs are currently ongoing (NCT02492230, NCT02606552).

### Limitations

Our study has several limitations. First, this study has all the inherent limitations of small retrospective study. Sample calculation was not possible because this study was a retrospective study dealing with a topic that had not been studied previously. Also, different cohorts of patients were derived from different time periods. Thus, the LAAO group had higher cerebrovascular risk, but had shorter follow-up for events to accrue. To compensate different follow-up period between the two groups, we analyzed and provided observation data up to 2 years. Second, the MAT group was highly disparate, and the duration of TAT was longer than suggested by the current guidelines. The diversity of antithrombotic regimens and variable duration of antithrombotic therapy, and low TTR might significantly affect clinical outcomes in present study. However, our findings might better reflect real-world practice and underscore the difficulty of following treatment guidelines. Third, in comparison between the NAT group and the LAAO group, the number of subjects in both groups was relatively small compared to the number of clinical events. Therefore, it should not be generalized that there is no difference in clinical outcomes between the NOAC-based antithrombotic strategy and the LAAO with DAPT strategy and should be reconfirmed in larger scale studies. Fourth, LAAO device failure was not included in study endpoints, since the purpose of this study was to prove that the bleeding event as well as the ischemic event can be reduced by performing the LAAO procedure. Fifth, data on coronary artery disease complexity such as SYNTAX score were not collected in this study. Sixth, only Koreans were included in this study. Therefore, it should be decided carefully to apply the results of this study to other ethnicities.

## Conclusions

LAAO with DAPT results in better net clinical outcomes in terms of preventing CVA and major bleeding than the MAT in patients with AF undergoing DES implantation. Further large-scale trials including comparisons with NOACs are warranted.

## Supporting information

S1 Fig Kaplan Meier curve. Freedom from clinical outcomes between the left atrial appendage occlusion group and the triple antithrombotic therapy subgroup during 24 months of follow-up. A: Rates of composite of cerebrovascular accidents (CVA) and major bleeding; B: Rates of CVA; C: Rates of major bleeding; D: Rates of major adverse cardiac and cerebral events (MACCE); E: Rates of cardiovascular death; F: Rates of all-cause death.
(TIF)

S2 Fig Kaplan Meier curve. Freedom from clinical outcomes between the left atrial appendage occlusion group and the non-triple antithrombotic therapy subgroup during 24 months of follow-up. A: Rates of composite of cerebrovascular accidents (CVA) and major bleeding; B: Rates of CVA; C: Rates of major bleeding; D: Rates of major adverse cardiac and cerebral events (MACCE); E: Rates of cardiovascular death; F: Rates of all-cause death.
(TIF)

S3 Fig Kaplan Meier curve. Freedom from clinical outcomes between the left atrial appendage occlusion group and the new oral anticoagulant (NOAC)-based antithrombotic therapy group during 24 months of follow-up. A: Rates of composite of cerebrovascular accidents (CVA) and major bleeding; B: Rates of CVA; C: Rates of major bleeding; D: Rates of major adverse cardiac and cerebral events (MACCE); E: Rates of cardiovascular death; F: Rates of all-cause death.
(TIF)

S4 Fig Expected and observed cerebrovascular accidents (CVA) and bleeding events between the left atrial appendage occlusion group and the non-triple antithrombotic therapy subgroup. Expected rates of CVA (A) and bleeding events (B) based on CHA2DS2-VASc and HAS-BLED scores were compared with observed event rates.
(TIF)

S1 Dataset (XLSX)

S1 File (DOCX)
