# Supplementary figures and images for: Comparison of clinical outcomes between multiple antithrombotic therapy versus left atrial appendage occlusion with dual antiplatelet therapy in patients with atrial fibrillation undergoing drug-eluting stent implantation
Source: PLoS One. 2021 Jan 7;16(1):e0244723. doi: 10.1371/journal.pone.0244723 (PMC7790384; doi:10.1371/journal.pone.0244723)

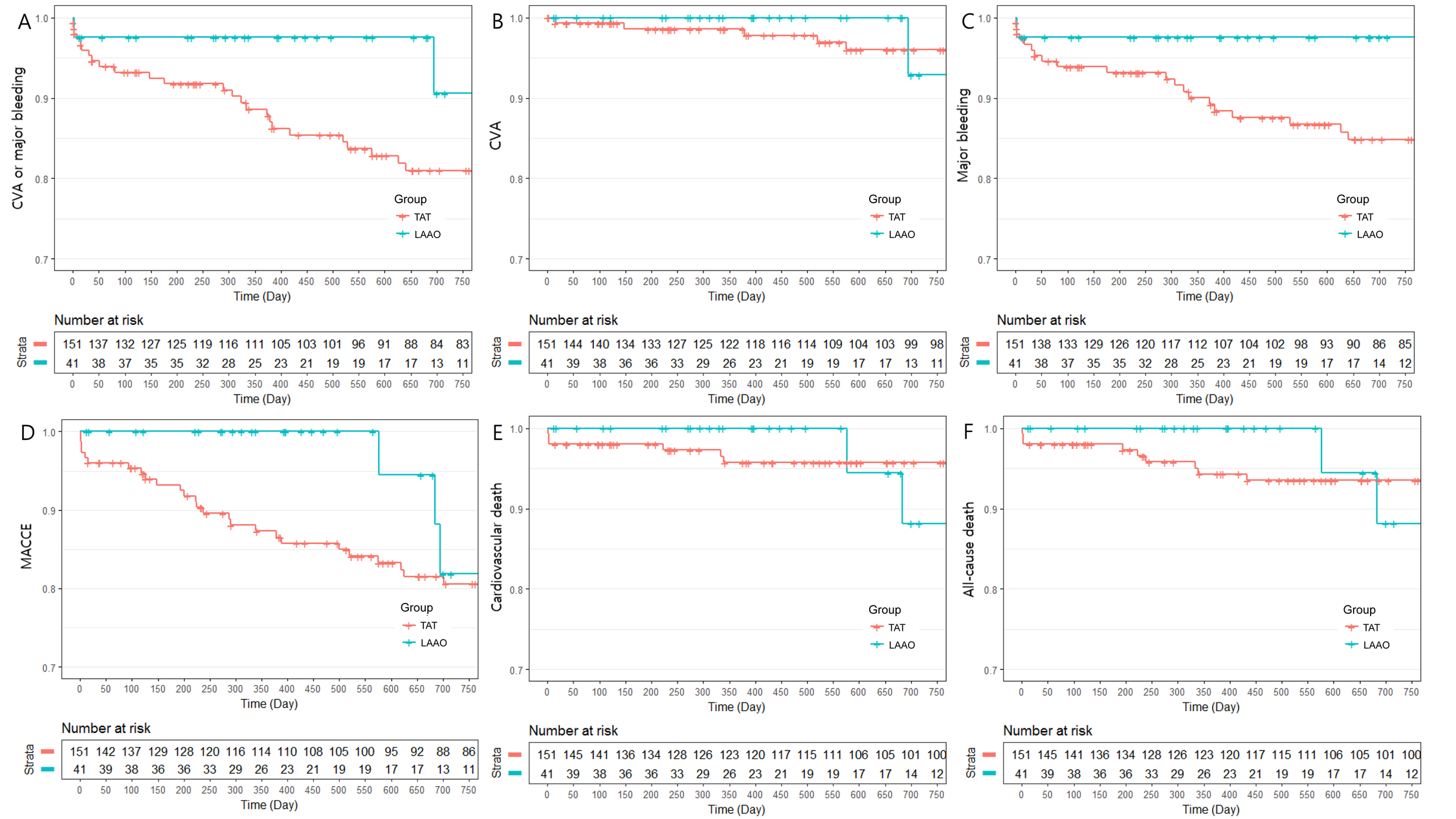

Supplement: S1 Fig — Freedom from clinical outcomes between the left atrial appendage occlusion group and the triple antithrombotic therapy subgroup during 24 months of follow-up. A: Rates of composite of cerebrovascular accidents (CVA) and major bleeding; B: Rates of CVA; C: Rates of major bleeding; D: Rates of major adverse cardiac and cerebral events (MACCE); E: Rates of cardiovascular death; F: Rates of all-cause death. (TIF) [file pone.0244723.s001.tif]

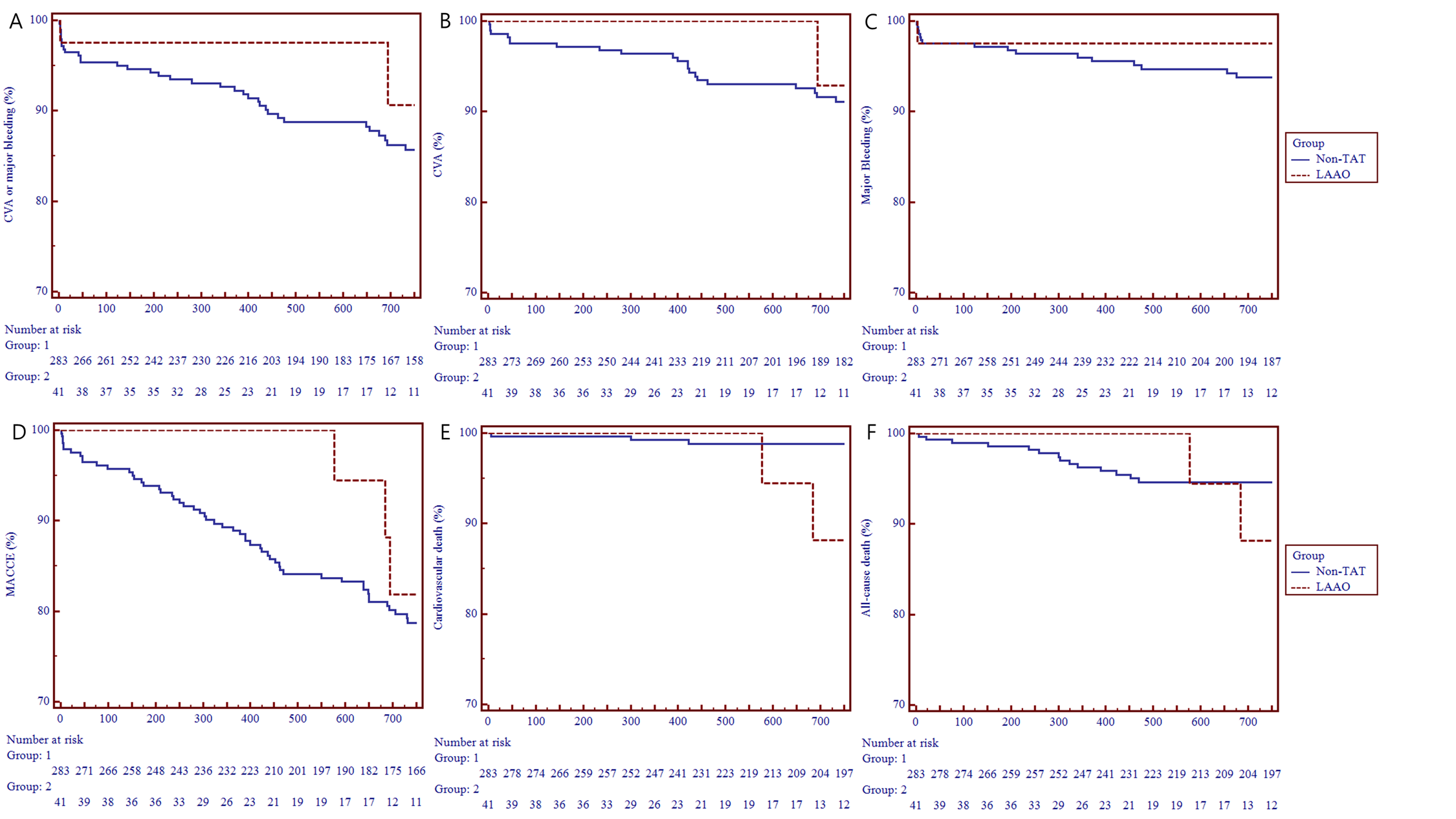

Supplement: S2 Fig — Freedom from clinical outcomes between the left atrial appendage occlusion group and the non-triple antithrombotic therapy subgroup during 24 months of follow-up. A: Rates of composite of cerebrovascular accidents (CVA) and major bleeding; B: Rates of CVA; C: Rates of major bleeding; D: Rates of major adverse cardiac and cerebral events (MACCE); E: Rates of cardiovascular death; F: Rates of all-cause death. (TIF) [file pone.0244723.s002.tif]

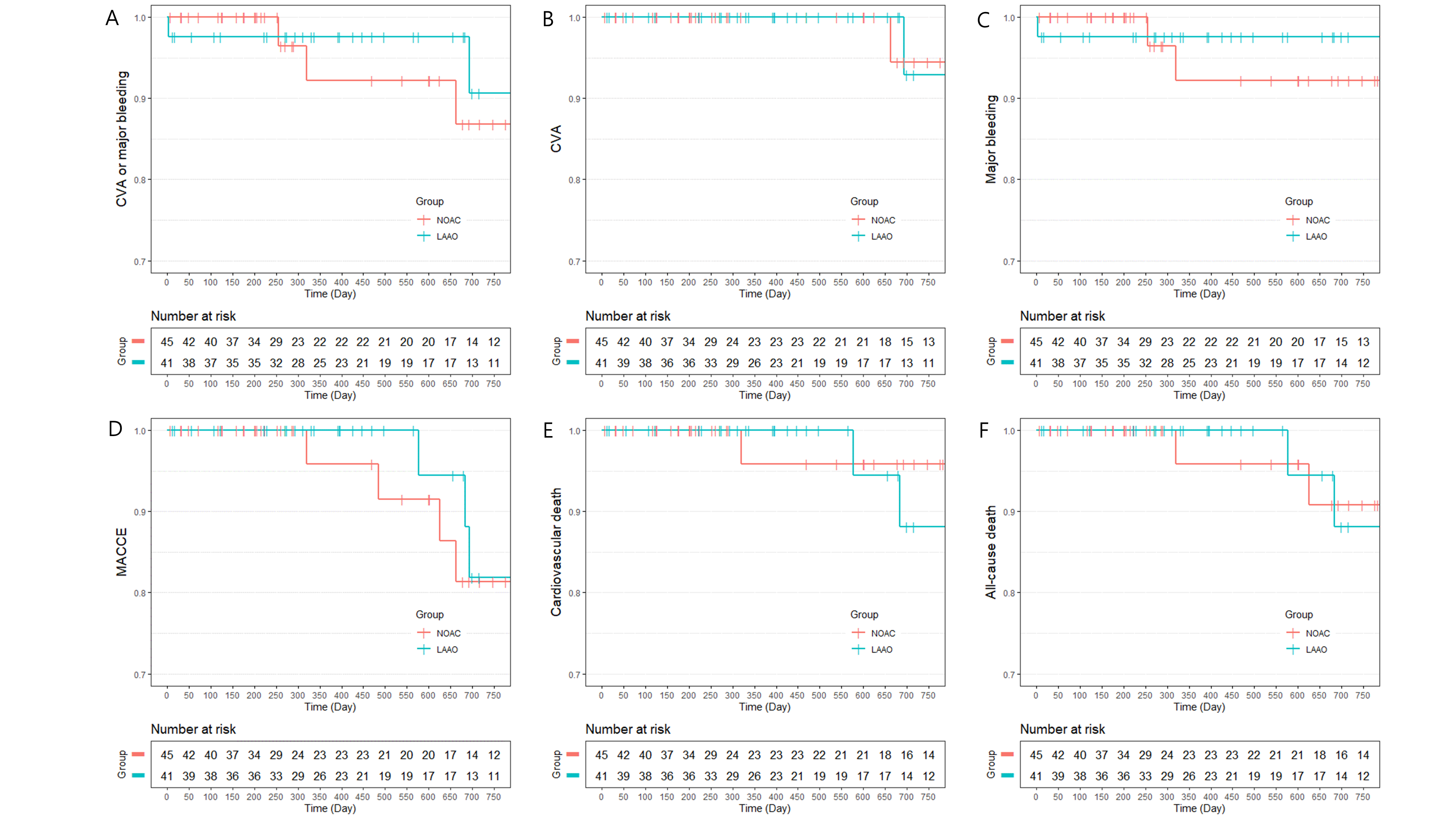

Supplement: S3 Fig — Freedom from clinical outcomes between the left atrial appendage occlusion group and the new oral anticoagulant (NOAC)-based antithrombotic therapy group during 24 months of follow-up. A: Rates of composite of cerebrovascular accidents (CVA) and major bleeding; B: Rates of CVA; C: Rates of major bleeding; D: Rates of major adverse cardiac and cerebral events (MACCE); E: Rates of cardiovascular death; F: Rates of all-cause death. (TIF) [file pone.0244723.s003.tif]

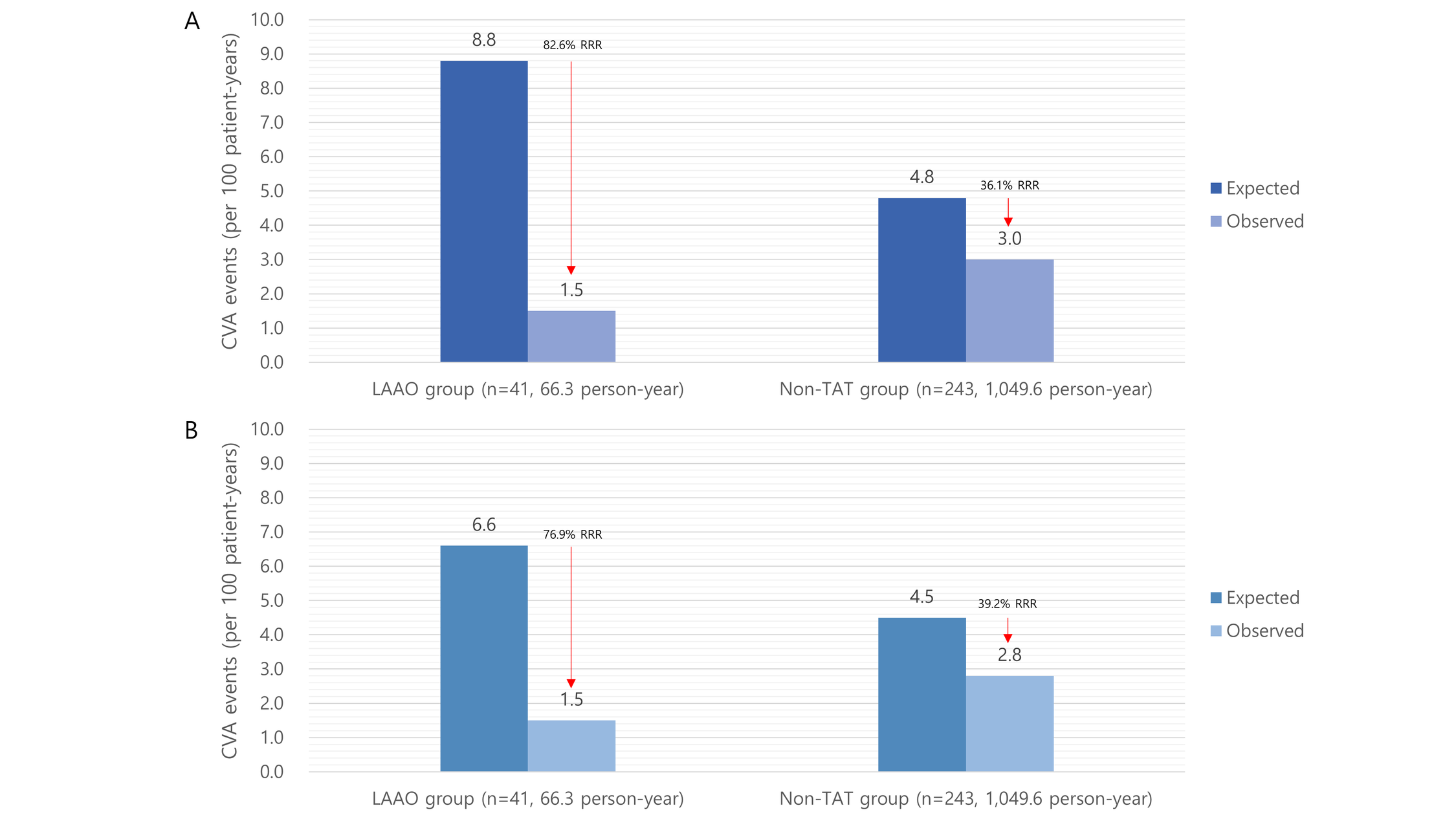

Supplement: S4 Fig — Expected rates of CVA (A) and bleeding events (B) based on CHA2DS2-VASc and HAS-BLED scores were compared with observed event rates. (TIF) [file pone.0244723.s004.tif]
