# Supplementary material for: Comparison of clinical outcomes between multiple antithrombotic therapy versus left atrial appendage occlusion with dual antiplatelet therapy in patients with atrial fibrillation undergoing drug-eluting stent implantation
Source: PLoS One. 2021 Jan 7;16(1):e0244723. doi: 10.1371/journal.pone.0244723 (PMC7790384; doi:10.1371/journal.pone.0244723)
Supplement: S1 File — (DOCX) [file pone.0244723.s006.docx]

**Supporting Methods**

This study was analyzed using SAS, SPSS Statistics and R. The process of generating propensity scores in R was as follows:

library(WeightIt)

library(ipw)

library(CBPS)

library(survival)

#df1 : the name of study data set

sdf1<- subset(df1, select=c(No, LAAO, age, SEXF1, smoking, hyperlipidemia, HTN, DM, CHF, spTIAorstroke, EF, Af, stentN, Diameter, TotallesionN))

covs0 <- subset(sdf1, select=-c(No, LAAO))

sdf1$p.score <- glm(f.build("LAAO", covs0), data = sdf1, family = "binomial")$fitted.values

df1$att.weights <- with(sdf1, LAAO + (1-LAAO)*p.score/(1-p.score))

coxph(Surv(CStrokeMBday1, StrokeMB1)~(LAAO+ smoking+DM+EF+stentN+Length, weights=att.weights, data=df1)

**Supporting Figure Legends**

**S1 Fig. Kaplan Meier curve.** Freedom from clinical outcomes between the left atrial appendage occlusion group and the triple antithrombotic therapy subgroup during 24 months of follow-up.

A: Rates of composite of cerebrovascular accidents (CVA) and major bleeding; B: Rates of CVA; C: Rates of major bleeding; D: Rates of major adverse cardiac and cerebral events (MACCE); E: Rates of cardiovascular death; F: Rates of all-cause death.

**S2 Fig. Kaplan Meier curve.** Freedom from clinical outcomes between the left atrial appendage occlusion group and the non-triple antithrombotic therapy subgroup during 24 months of follow-up.

A: Rates of composite of cerebrovascular accidents (CVA) and major bleeding; B: Rates of CVA; C: Rates of major bleeding; D: Rates of major adverse cardiac and cerebral events (MACCE); E: Rates of cardiovascular death; F: Rates of all-cause death.

**S3 Fig. Kaplan Meier curve.** Freedom from clinical outcomes between the left atrial appendage occlusion group and the new oral anticoagulant (NOAC)-based antithrombotic therapy group during 24 months of follow-up.

A: Rates of composite of cerebrovascular accidents (CVA) and major bleeding; B: Rates of CVA; C: Rates of major bleeding; D: Rates of major adverse cardiac and cerebral events (MACCE); E: Rates of cardiovascular death; F: Rates of all-cause death.

**S4 Fig. Expected and observed cerebrovascular accidents (CVA) and bleeding events between the left atrial appendage occlusion group and the non-triple antithrombotic therapy subgroup.** Expected rates of CVA (A) and bleeding events (B) based on CHA2DS2-VASc and HAS-BLED scores were compared with observed event rates.

**Supporting Tables**

**S1 Table. Inverse probability of treatment weighting-adjusted baseline characteristics of the study population**

|  | LAAO group | MAT group | P-value |
| --- | --- | --- | --- |
| Age (years) | 67.6±9.4 | 68.1±8.6 | 0.76 |
| Male | 71.6 | 67.0 | 0.576 |
| Smoking |  |  | 0.051 |
| Never | 73.3 | 61.1 |  |
| Ex-smoker | 11.1 | 24.1 |  |
| Current-smoker | 15.6 | 14.8 |  |
| HTN | 70.9 | 75.4 | 0.579 |
| DM | 47.5 | 31.6 | 0.03 |
| CHF | 38.1 | 29.0 | 0.224 |
| Dyslipidemia | 57.9 | 46.9 | 0.153 |
| Previous CVA | 25.1 | 17.1 | 0.225 |
| EF (%) | 55.3±9.7 | 52.1±13.6 | 0.06 |
| Lesion number | 2.1±0.8 | 2.1±1.1 | 0.743 |
| Stent number | 1.3 ± 0.5 | 1.5 ± 0.8 | 0.015 |
| Diameter (mm) | 3.0 ± 0.4 | 3.0 ± 0.4 | 0.983 |
| Length (mm) | 22.3±7.9 | 35.4±22.3 | <0.0001 |
| AF type |  |  | 0.325 |
| Paroxysmal | 20.9 | 27.8 |  |
| Persistent or permanent | 79.1 | 72.2 |  |

Values are mean ± SD or n (%). AF = atrial fibrillation; CHF = congestive heart failure; CVA = cerebrovascular accidents; DM = diabetes mellitus; EF = ejection fraction; HTN = hypertension; LAAO = left atrial appendage occlusion; MAT = multiple antithrombotic therapy

**S2 Table. Baseline characteristics comparison between LAAO group and NOAC-based antithrombotic therapy group.**

|  | LAAO  (n=41) | NOAC  (n=45) | P-value |
| --- | --- | --- | --- |
| Age (years) | 69.6±8.3 | 73.9±9.7 | 0.021 |
| Male | 32 (78.1) | 36 (80.0%) | 0.824 |
| Follow-up period (days) | 590.5±420.3 | 571.7±502.9 | 0.852 |
| Smoking |  |  | 0.312 |
| Never | 36 (87.8) | 37 (82.2) |  |
| Ex-smoker | 3 (7.3) | 1 (2.2) |  |
| Current smoker | 2 (4.9) | 7 (15.6) |  |
| HTN | 29 (70.7) | 38 (84.4) | 0.126 |
| DM | 20 (48.8) | 17 (37.8) | 0.303 |
| CHF | 15 (36.6) | 8 (17.8) | 0.049 |
| Dyslipidemia | 24 (58.5) | 17 (37.8) | 0.054 |
| Previous CVA | 18 (43.9) | 1 (2.2) | <0.001 |
| CHADS2 score | 2.71±1.27 | 1.91±1.02 | 0.004 |
| CHA2DS2-VASc score | 4.56±1.55 | 3.24±1.43 | <0.001 |
| CHA2DS2-VASc≥2 | 41 (100.0) | 39 (86.7) | 0.027 |
| HAS-BLED score | 3.24±1.20 | 2.20±0.87 | <0.001 |
| EF (%) | 54.35±11.01 | 47.80±13.83 | 0.088 |
| Lesion number | 2.00±0.86 | 1.69±0.79 | 0.169 |
| Stent number | 1.28±0.51 | 1.33±0.64 | 0.772 |
| Diameter (mm) | 3.14±0.50 | 3.16±0.54 | 0.919 |
| Length (mm) | 22.00±7.78 | 25.63±12.56 | 0.184 |
| AF type |  |  | 0.182 |
| Paroxysmal | 10 (24.4) | 17 (37.8) |  |
| Persistent or permanent | 31 (75.6) | 28 (62.2) |  |

Values are mean ± SD or n (%). AF = atrial fibrillation; CHF = congestive heart failure; CVA = cerebrovascular accidents; DM = diabetes mellitus; EF = ejection fraction; HTN = hypertension; LAAO = left atrial appendage occlusion; NOAC = new oral anticoagulants

**S3 Table. Composition of the NOAC-based antithrombotic therapy group.**

| Variables | n=45 |
| --- | --- |
| Triple antithrombotic therapy | 36 (80.0) |
| Duration (Days) | 120.1±147.9 |
| De-escalation after triple antithrombotic therapy |  |
| Not done | 2 (5.6) |
| Double antithrombotic therapy (NOAC+aspirin) | 5 (13.9) |
| Double antithrombotic therapy (NOAC+P2Y12 inhibitor) | 24 (66.7) |
| NOAC alone | 4 (11.1) |
| Dual antiplatelet therapy | 1 (2.8) |
| Double antithrombotic therapy | 7 (15.6) |
| Duration (Days) | 568.6±162.4 |
| De-escalation after dual antithrombotic therapy |  |
| Not done | 4 (57.1) |
| NOAC alone | 2 (28.6) |
| Single antiplatelet therapy | 1 (14.3) |
| Dual antiplatelet therapy | 2 (4.4) |

Values are mean ± SD or n (%).NOAC = new oral anticoagulant

**S4 Table. Inverse probability of treatment weighting-adjusted clinical outcomes between the LAAO group and the TAT subgroup at 24 months of follow-up.**

| Variables | LAAO  (n=41) | TAT  (n=151) | Log-rank P-value | Hazard ratio  (95% Confidence interval) | P-value |
| --- | --- | --- | --- | --- | --- |
| CVA or major bleeding | 2 (9.4%) | 25 (19.0%) | 0.113 | 0.34 (0.146 – 0.795) | 0.013 |
| CVA | 1 (7.1%) | 5 (4.0%) | 0.991 | 2.033 (0.529 – 7.81) | 0.302 |
| Major bleeding | 1 (2.4%) | 20 (15.2%) | 0.0842 | 0.113 (0.027 – 0.479) | 0.003 |
| Myocardial infarction | 1 (5.6%) | 4 (2.9%) | 0.9754 | 0.38 (0.052 – 2.78) | 0.341 |
| MACCE | 3 (18.1%) | 26 (19.4%) | 0.232 | 0.819 (0.385 – 1.744) | 0.605 |
| Cardiovascular death | 2 (11.9%) | 6 (4.3%) | 0.624 | 4.354 (1.146 – 16.538) | 0.031 |
| All-cause death | 2 (11.9%) | 9 (6.5%) | 0.979 | 1.877 (0.591 – 5.964) | 0.285 |

Data shown are number of patients (%). Event rates were Kaplan-Meier estimates. Adjusted variables for Cox analysis were smoking, diabetes mellitus, ejection fraction, stent number, and stent length. CVA = cerebrovascular accidents; LAAO = left atrial appendage occlusion; MACCE = major adverse cardiac and cerebral event; TAT = triple antithrombotic therapy

**S5 Table. Cumulative incidence of clinical outcomes between the LAAO group and the non-TAT subgroup at 24 months of follow-up.**

| Variables | LAAO  (n=41) | Non-TAT  (n=283) | Log-rank P-value |
| --- | --- | --- | --- |
| CVA or major bleeding | 2 (9.4%) | 34 (13.8%) | 0.188 |
| CVA | 1 (7.1%) | 21 (8.4%) | 0.316 |
| Major bleeding | 1 (2.4%) | 16 (6.2%) | 0.384 |
| MACCE | 3 (18.1%) | 53 (20.8%) | 0.101 |
| Cardiovascular death | 2 (11.9%) | 3 (1.2%) | 0.017 |
| All-cause death | 2 (11.9%) | 14 (5.4%) | 0.990 |

Data shown are number of patients (%). Event rates were Kaplan-Meier estimates. CVA = cerebrovascular accidents; LAAO = left atrial appendage occlusion; MACCE = major adverse cardiac and cerebral event; TAT = triple antithrombotic therapy

**S6 Table. Inverse probability of treatment weighting-adjusted clinical outcomes between the LAAO group and the NOAC-based antithrombotic therapy group.**

| Variables | LAAO  (n=41) | NOAC  (n=45) | Hazard ratio  (95% Confidence interval) | P-value |
| --- | --- | --- | --- | --- |
| CVA or major bleeding | 2 (9.4%) | 4 (42.1%) | 1.03 (0.67 – 1.57) | 0.897 |
| CVA | 1 (7.1%) | 1 (5.6%) | 1.00 (0.65 – 1.52) | 0.991 |
| Major bleeding | 1 (2.4%) | 3 (38.5%) | 1.03 (0.67 – 1.58) | 0.888 |
| Myocardial infarction | 1 (5.6%) | 1 (4.5%) | 0.96 (0.63 – 1.47) | 0.853 |
| MACCE | 3 (18.1%) | 5 (26.8%) | 0.99 (0.65 – 1.51) | 0.959 |
| Cardiovascular death | 2 (11.9%) | 2 (12.9%) | 0.96 (0.63 – 1.46) | 0.836 |
| All-cause death | 2 (11.9%) | 3 (17.5%) | 0.97 (0.64 – 1.49) | 0.899 |

Data shown are number of patients (%). Event rates were Kaplan-Meier estimates. Adjusted variables for Cox analysis were smoking, diabetes mellitus, ejection fraction, stent number, and stent length. CVA = cerebrovascular accidents; LAAO = left atrial appendage occlusion; MACCE = major adverse cardiac and cerebral event; NOAC = new oral anticoagulant
